# Seroprevalence of *Leptospira* Hardjo in the Irish suckler cattle population

**DOI:** 10.1186/2046-0481-65-8

**Published:** 2012-04-30

**Authors:** Eoin Gerard Ryan, Nola Leonard, Luke O’Grady, Simon J More, Michael L Doherty

**Affiliations:** 1 School of Veterinary Medicine, University College Dublin, Belfield, Dublin 4, Republic of Ireland

**Keywords:** Leptospirosis, Hardjo, Suckler, Ireland, Seroprevalence, ELISA, Herd size, Region, FreeCalc, Endemic

## Abstract

**Background:**

Prior to the present study, the seroprevalence of leptospirosis in Irish suckler herds was unknown. In this study, we describe the herd and animal-level prevalence of *Leptospira* Hardjo infection in the Irish suckler cattle population. For the purposes of the study, the 26 counties of the Republic of Ireland were divided into 6 regions from which a representative number of herds were selected. A herd was considered eligible for sampling if it was not vaccinating against leptospirosis and if it contained ≥ 9 breeding animals of beef breed ≥ 12 months of age. In total, 288 randomly selected herds were eligible for inclusion in the seroprevalence dataset analysis. Serological testing was carried out using a commercially available monoclonal antibody-capture ELISA, (sensitivity 100%; specificity 86.67%).

**Results:**

Herds were categorised as either “Free from Infection” or “Infected” using the epidemiological software tool, *FreeCalc 2.0*. Using this classification, 237 herds were “Infected” (82.29%). The South West and South East regions had the highest herd prevalence. The regional effect on herd prevalence was largely mirrored by breeding herd size. A true animal-level prevalence of 41.75% was calculated using the epidemiological software tool, *TruePrev*. There was a statistically significant regional trend, with true prevalence being highest in the South East (*P* < 0.05). The median Breeding Herd Size (BHS), when categorised into quartiles, had a statistically significant influence on individual animal true seroprevalence (*P* < 0.001); true seroprevalence increased with increasing BHS.

**Conclusions:**

Leptospirosis is a widespread endemic disease in the Republic of Ireland. It is possible that economic losses due to leptospirosis in unvaccinated Irish suckler herds may be underestimated.

## Background

Leptospirosis is a well recognised disease of cattle worldwide [1-7]. Two species of leptospires are associated with the disease: *Leptospira interrogans* serovar Hardjo and *Leptospira borgpetersenii* serovar Hardjo*.* Whilst there are genetic, epidemiological and pathogenic differences between the two species, the two microorganisms are indistinguishable by serological tests [8-10]. Collectively, both species can be referred to as *Leptospira* Hardjo. *Leptospira* Hardjo mainly causes reproductive disease, i.e. abortion, mummification, stillbirth, premature and term birth of weak calves [11-14], as well as causing milk drop syndrome in dairy herds [15,16]. Cattle act as a maintenance host for *Leptospira* Hardjo [17], and shed leptospires in both urine and discharges from the genital tract [18-23]. Leptospirosis is recognised as a significant zoonotic disease of farmers, farm workers and workers involved in the agricultural industry worldwide [24-27].

Leptospirosis due to *Leptospira* Hardjo is recognised as a cause of clinical disease in cattle in the Republic of Ireland and Northern Ireland [14,28]. In a more recent study of unvaccinated Irish dairy herds, 79% had a positive bulk tank milk ELISA titre to Leptospira Hardjo [29]. Prior to the present study, the seroprevalence of leptospirosis, and associated risk factors, in Irish suckler herds were unknown.

In this study, we describe the herd and animal-level prevalence of *Leptospira* Hardjo infection in the suckler cattle population in the Republic of Ireland. Herd-level results are presented by area (region and county) and breeding herd size, and animal-level results by area, breeding herd size, age and sex.

## Materials and methods

### Study design

This seroprevalence study was conducted using a cross-sectional study design, in conjunction with a national survey to estimate the prevalence of paratuberculosis in Ireland [30]. With permission from officials of the Department of Agriculture, Food & Fisheries, serum samples were selected in 2005 and 2006 at the Central Regional Veterinary Laboratory, Abbotstown, Co. Dublin. A list of herds and individual animals displaying their tag numbers, age, sex and breed was available. This population of herds was a subset of the national herd as chosen randomly from the herds tested for brucellosis in 2004 and 2005 under the National Brucellosis Eradication Scheme. They consisted of 1,000 herds (mixed suckler and dairy) randomly chosen from an eligible total of 96,163 herds where at least one calf had been registered on the Cattle Movement Monitoring System (CMMS) as born in the herd in 2003 [31]. One ml of serum was collected for each animal included in the study and transported to the Veterinary Sciences Centre, UCD for laboratory analysis. Samples were frozen at-20°C between collection and ELISA testing.

### Study population

The reference population consisted of the 1,000 herds (mixed suckler and dairy). A random sample of suckler herds was then chosen from among this mixed subset of the population. The individual animal eligibility criteria were unvaccinated females and bulls of beef breeds ≥ 12 months of age. Only herds with ≥ 9 eligible animals were included in the study.

Assuming a herd seroprevalence of 70% in Irish suckler herds (83,630 herds), in accordance with results in Irish and UK herds (Leonard et al., 2004; Pritchard, 1987), the number of herds required for sampling, to estimate the prevalence of leptospirosis to within 5% at the 95% confidence level was 320 [32]. The number of herds and cows in the Irish suckler population was taken from data in the Central Statistics Office Census of Agriculture, 2000 [33].

**Figure 1:**
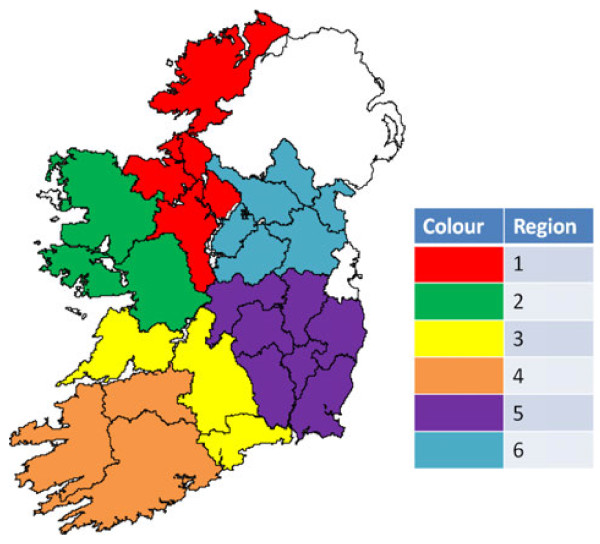
**Regions in the Republic of Ireland used in the *Leptospira*Hardjo seroprevalence study.**

The 26 counties of the Republic of Ireland were divided into 6 regions (Region 1 – North West; Region 2 – West Connaught; Region 3 – North Munster; Region 4 – South West Munster; Region 5 – South East Leinster; Region 6 – North Leinster/South Ulster) based on broadly similar husbandry practices and farmland type, with each region containing approximately 200,000 suckler cows (Figure1).

The number of herds sampled in each region was proportionate to the percentage of the national herd made up by the herds in that region (Table1).

**Table 1:** The number of suckler herds required for sampling (proportional sample) per region in a Leptospiral seroprevalence study in proportion to the percentage of the National Suckler Herd made up by herds in that region

	**Suckler cow population**	**Number of herds**	**Total Herds % of National Suckler Herd**	**Proportional Sample Required Number of herds**
**Region 1**	185,665	15,880	19.0	**61**
**Region 2**	202,787	18,140	21.7	**71**
**Region 3**	186,928	10,470	12.5	**40**
**Region 4**	197,100	13,970	16.7	**54**
**Region 5**	208,816	11,880	14.2	**46**
**Region 6**	205,693	13,290	15.9	**51**

### ELISA

The *Linnodee Leptospira ELISA Kit*^TM^ (Linnodee Animal Care, Ballyclare, Northern Ireland) [34,35] was used to test all serum samples. This ELISA detects an antibody response to a lipopolysaccharide outer envelope epitope common to both *Leptospira borgpetersenii* serovar Hardjo and *Leptospira interrogans* serovar Hardjo [35]. This was a double sandwich ELISA in which Hardjo antigen was bound to wells pre-coated with Hardjo lipopolysaccharide specific monoclonal antibody. This Hardjo antigen reacted with Hardjo-specific antibodies in the diluted bovine sera after it was transferred to the plate wells. Positive and negative sera controls were provided with the ELISA kit and three replicates of each control were used per plate. The immobilised Hardjo antibodies were then detected by the addition of peroxidase conjugated antibody. This was then quantified by measuring the amount of labelled detection antibody bound to the matrix using a chromogenic substrate (TMB). The plate was read using a molecular devices *VersaMax *^TM^ microplate reader (Associates of Cape Cod Incorporated, Massachusetts, USA) at a wavelength of 450 nm. The test was assumed to have a sensitivity and specificity of 100% and 86.67%, respectively, as reported previously [34].

### Questionnaire

A questionnaire was posted to all herds initially chosen for the study (320 herds), to determine the vaccination status of each herd, and to collect epidemiological data on potential risk factors for herd seropositivity to *Leptospira* Hardjo in Irish suckler herds. The results of the questionnaire survey will be discussed in a separate paper on the risk factors for *Leptospira* Hardjo in Irish beef/suckler herds. The herdowners that did not return the questionnaire were contacted by telephone to determine whether they were vaccinating against leptospirosis and, thus, whether they were eligible for inclusion in the study.

### Variables

In this study, the key measures of interest included herd- and animal-level seroprevalence. The results were presented by area (region and county) and breeding herd size (for herd- and animal-level prevalence), and also by age and sex (animal-level prevalence only).

### Herd-level seroprevalence

Herd-level seroprevalence was determined after first defining each study herd as “infected” or not, based on the serological results obtained. A programme, *FreeCalc 2.0*[36-38], was used during herd classification, calculating the probability of freedom from infection in each study herd, given the test results, the likely minimum herd prevalence assuming infection, the limitations of the serological test (in particular, imperfect specificity leading to false positive results) and after accounting for finite herd size. The methodology is a probabilistic approach to this problem, with the application of a hypergeometric exact probability formula and a result expressed in terms of probability of freedom. The following data were used during these calculations: test (ELISA) sensitivity and specificity, estimated minimum expected (within-herd) infection prevalence, and population (herd) size. Herd-level sensitivity (HSENS) and herd-level specificity (HSPEC) were chosen to be 95% respectively. Based on knowledge of the biology of the disease [39], on published within-herd prevalence rates in endemic herds (62% [40]; 41.8% [41]), and using a trial and error approach, it was found that a within-herd prevalence of 40% allowed the rejection of the null hypothesis (null hypothesis = herds are infected) when sampling a maximum of 20 animals per herd, using the ELISA with test sensitivity of 100% and test specificity of 86.67%. For herds of < 20 eligible breeding animals, all animals were sampled. Ultimately, all herds were classified as either “Free from Infection” or “Infected” at the 95% confidence interval at a within-herd prevalence of 40%.

### Animal-level seroprevalence

Individual animal seroprevalence was determined by the *Linnodee Bovine Leptospirosis Kit*^TM^ ELISA (LLK). A sample was considered positive if the Ratio > Negative Cut-off where:

(1) Ratio=(sample OD/mean positive control OD)

(2) Negative Cut−off=(mean negative control OD/mean positive control OD)×2

Where OD = optical density.

The apparent prevalence within each herd was calculated by expressing the number of ELISA-positive animals as a percentage of the total number of animals sampled in the herd. Estimated true within-herd prevalence, at the 95% confidence interval, was then calculated by using the epidemiological computer software tool, *TruePrev*[42], which takes into account the sensitivity and specificity of the test used and the number of animals tested.

### Statistical methods

#### Data management

Data were managed using Microsoft Excel (Microsoft Office 2007, Microsoft Corporation, Redmond, Washington, USA) and processed using PASW Statistics 18 (SPSS Inc., Chicago, USA).

#### Data analysis

The one-way ANOVA test was used to assess the relationship between median breeding herd size, divided by quartiles, and within-herd true prevalence. For other associations, the lack of overlap of relevant confidence intervals was used to demonstrate statistical significance.

## Results

### Study population

The total number of herds from which serum samples were obtained was 320 (Table2), of which, vaccination was practised in 21 herds and 11 herds were ineligible due to inadequate size, leaving a total of 288 herds eligible for inclusion in the study. Herds were sampled in all counties except Dublin.

**Table 2:** Summary information for herds sampled in each of six regions in a Leptospiral seroprevalence survey

	**Target Sample number**	**Actual number of herds sampled**	**Number of animals tested**	**Herds vaccinating**	**Herds ≤ 8 breeding animals**	**Number of herds eligible**
**Region 1**	61	65	1011	3	4	58
**Region 2**	71	63	1018	6	3	54
**Region 3**	40	51	918	1	0	50
**Region 4**	54	51	867	3	1	47
**Region 5**	46	50	888	4	1	45
**Region 6**	51	40	664	4	2	34
**Totals**	323	320	5366	21	11	288

Table2 also summarises the number of individual animals that were tested in each region. In total, sera from 5,366 eligible animals were ELISA-tested.

### Descriptive data

#### Herd-level seroprevalence

Analysis of the results contained in Table3 reveal that 82.29% of the 288 herds sampled were classified as infected (HSENS & HSPEC of 95%). Herd prevalence varied from 75.93% in Region 2 herds to 93.33% in herds in Region 5.

**Table 3:** Herd prevalence (%) of Leptospiral infection by region in a Leptospiral seroprevalence study, with data on median breeding herd size (BHS) per region

	**Herds Free from Infection**	**Herds Infected**	**Total Herds**	**Herd Prevalence %**	**Median BHS**
Region 1	10	48	58	**82.76**	20.5
Region 2	13	41	54	**75.93**	19.5
Region 3	10	40	50	**80.00**	28.0
Region 4	7	40	47	**85.11**	21.0
Region 5	3	42	45	**93.33**	28.0
Region 6	8	26	34	**76.47**	23.5
**National**	**51**	**237**	**288**	**82.29**	**22.00**

**Figure 2:**
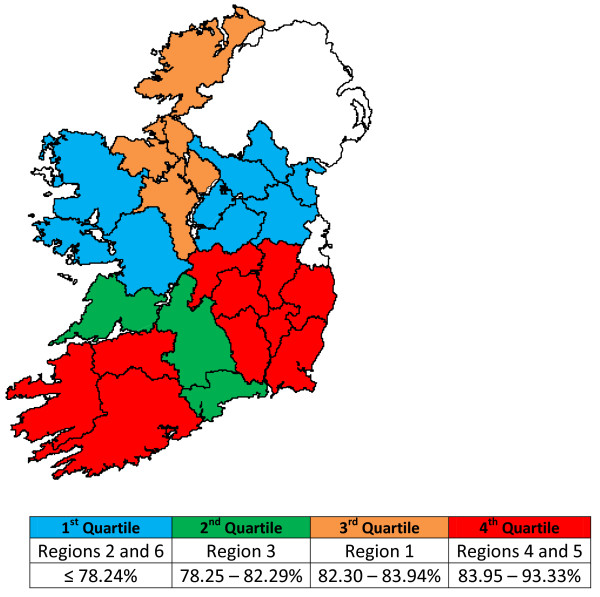
Herd prevalence (%) of Leptospiral infection by region with prevalence divided by quartiles.

Figure2 displays herd prevalence per Region by dividing the data into quartiles. This shows that South West Munster (Region 4) and South East Leinster (Region 5) had the highest herd prevalence, with West Connaught (Region 2) and North Leinster/South Ulster (Region 6) having the lowest herd prevalence.

**Figure 3:**
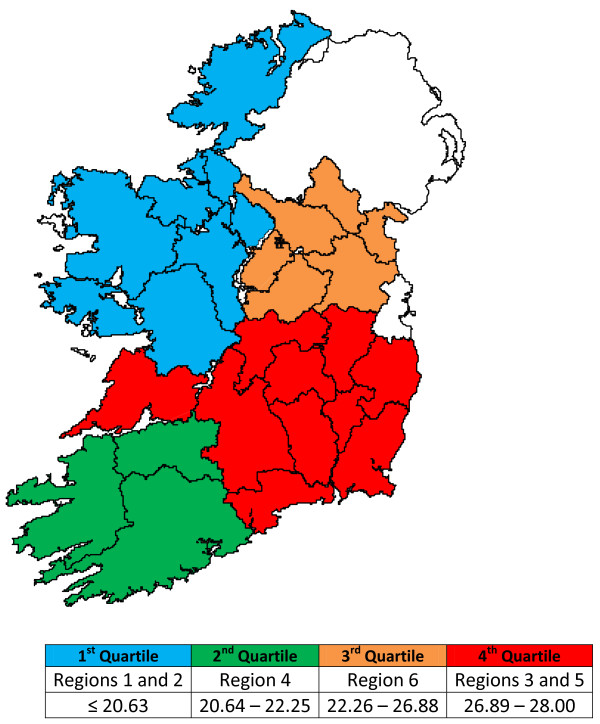
Median breeding herd size by region with herd size divided by quartiles.

Median Breeding Herd Size (BHS) for each Region is also presented in Table3. It can be seen that Regions 3 and 5 have the largest median BHS. The results for median BHS per region were divided by quartiles and mapped (Figure3). This figure clearly displays a regional effect with the largest herds occurring in the South East (Regions 3 and 5) of the country.

#### Seroprevalence data: Individual animal-level

**Figure 4:**
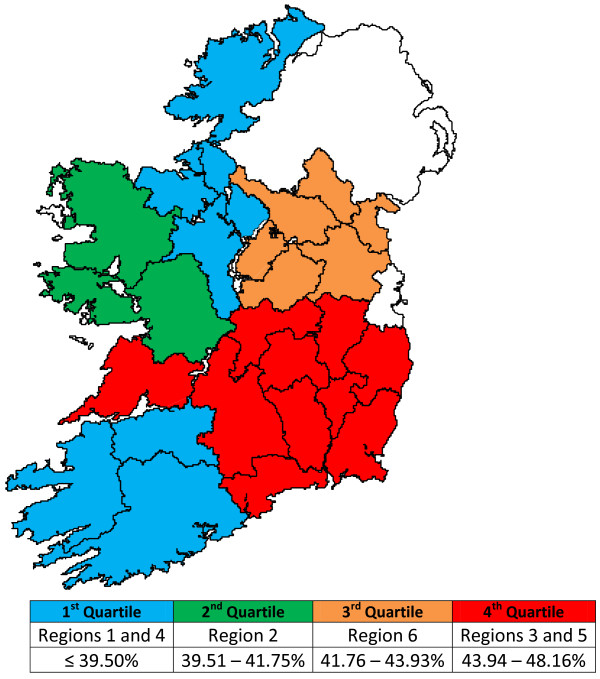
True seroprevalence (%) of Leptospiral infection in individual animals by region, with seroprevalence divided by quartiles.

The results of the individual animal ELISA testing are displayed by Region, Age, Sex and Breeding Herd Size in Table4. It can be seen that there is a true prevalence of 41.75% on a national level. True prevalence was highest in Region 5 at 48.16%, while Region 1 had the lowest true prevalence at 36.32%. As with the herd prevalence data, there is a regional trend, with true prevalence being highest in the South East (Region 3 and 5) of the country. This is illustrated in Figure4. When the 95% confidence intervals were examined, there were statistically significant differences in true prevalence between Region 1 and Regions 2, 3, 5 & 6 and between Region 2 and Regions 3 & 5 (*P* < 0.05).

**Table 4:** **The seroprevalence of*Leptospira*Hardjo in individual animals in a Leptospiral seroprevalence study: data presented by region, age, sex and breeding herd size**

**Category**		**ELISA Negative**	**ELISA Positive**	**Total Tested**	**Apparent Prevalence (%)**	**True Prevalence (%)**	**95% Confidence Interval**
**Region**	**1**	558	453	1011	44.81	36.32	34.67 - 37.96
	**2**	518	500	1018	49.12	41.29	39.64 - 42.93
	**3**	436	482	918	52.51	45.20	43.47 - 46.93
	**4**	468	399	867	46.02	37.72	35.94 - 39.49
	**5**	399	489	888	55.07	48.16	46.40 - 49.91
	**6**	330	334	664	50.30	42.66	40.62 - 44.69
**Age**	**Unknown**	29	37	66	56.06	49.30	42.88 - 55.71
	**1–2 yrs**	763	726	1489	48.76	40.88	39.51 - 42.23
	**2–3 yrs**	207	180	387	46.51	38.28	35.62 - 40.94
	**3–5 yrs**	776	777	1553	50.03	42.35	41.01 - 43.67
	**5–9 yrs**	527	527	1054	50.00	42.31	40.69 - 43.92
	**>9 yrs**	407	410	817	50.18	42.52	40.68 - 44.35
**Sex**	**Unknown**	29	37	66	56.06	49.30	42.88 - 55.71
	**Female**	2570	2514	5084	49.45	41.67	40.93 - 42.41
	**Male**	110	106	216	49.07	41.23	37.66 - 44.80
**Breeding Herd Size**	**≤13**	453	325	778	41.77	32.81	30.95 - 34.67
	**14–23**	824	709	1533	46.25	37.98	36.64 - 39.32
	**24–32**	624	572	1196	47.83	39.80	38.29 - 41.32
	**33–142**	808	1051	1859	56.54	49.85	48.64 - 51.06
ALL		2709	2657	5366	49.52	41.75	41.03 - 42.47

**Figure 5:**
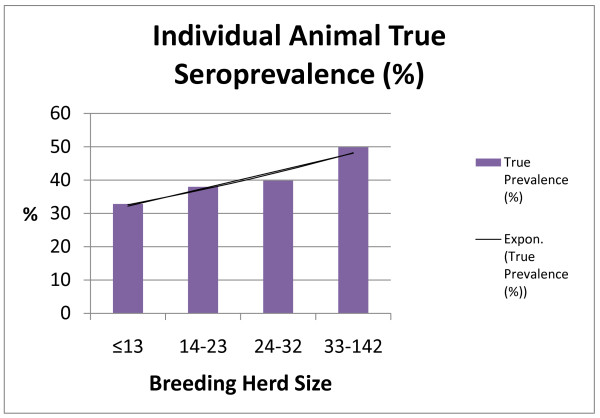
Influence of breeding herd size on true seroprevalence (%) of Leptospiral infection in individual animals.

To more accurately assess the influence of BHS on individual animal seroprevalence, BHS was divided by quartiles (Table4). There was a statistically significant influence of BHS on individual animal true seroprevalence; true seroprevalence increases with increasing BHS (Figure5). Statistical analysis carried out using the one-way ANOVA test showed that there was a statistically significant difference in within-herd prevalence between first quartile and fourth quartile herds, and between second quartile herds and fourth quartile herds (*P* < 0.001).

**Figure 6:**
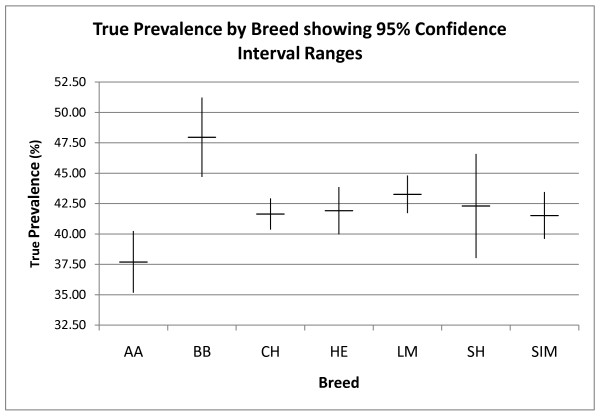
True seroprevalence (%) of Leptospiral infection by breed showing 95% confidence interval ranges.

The two most common breeds were Charolais (1,659) and Limousin (1,139). True seroprevalence by breed is displayed in Figure6. There were statistically significant differences in breed seroprevalence between Aberdeen Angus and Belgian Blue (*P* < 0.05); between Aberdeen Angus and Charolais (*P* < 0.05) and between Aberdeen Angus and Limousin (*P* < 0.05).

There was little variation in individual animal true seroprevalence according to age category or sex (Table4).

## Discussion

This was the first serological survey of Leptospiral infection due to *Leptospira* Hardjo in suckler cattle herds in the Republic of Ireland. The survey provides useful descriptive epidemiological data, because herds were distributed throughout the country (with the exception of Co. Dublin) and were sampled in proportion to the number of herds in each Region. Regional influences on seroprevalence have previously been shown to be important in dairy herds in Ireland [29].

## Key results

The key results to emerge from this study were the finding that there was an overall herd prevalence of 82.29% (HSENS & HSPEC of 95%), and an individual animal true seroprevalence of 41.75% nationally, indicating that leptospirosis is a widespread endemic disease in this country. There was also a notable regional variation in herd and individual animal prevalence, with the South-East having the highest prevalence of leptospirosis and the West having the lowest prevalence. This clear regional demarcation is mirrored closely by median Breeding Herd Size in these regions. The association between median breeding herd size, sorted by quartiles, and within-herd prevalence was found to be statistically significant (*P* < 0.001).

### Limitations of the study

While herds were chosen at random, they were chosen from a defined population, i.e. the paratuberculosis study herds, which may have led to a degree of bias. However, there was a wide distribution of herd sizes amongst the 320 selected herds and 25 counties were represented, with good proportionality between regions. The decision to apply a modified stratified sampling approach to the selection of eligible animals from herds of different sizes was made in order to remain within the budgetary constraints of the study and is a recognised approach to adopt in studies such as this [43]. From the point of view of classifying herds as “Free from Infection” or “Infected”, the software program, *FreeCalc 2.0*[36], allowed for the entry of each herd’s true breeding herd size, as well as the number of animals tested and the number of positive reactors, when calculating herd disease status.

The relative lack of specificity of the *Linnodee*^TM^ ELISA (LLK) (86.67%), when compared to the Microscopic Agglutination Test (MAT), could be considered a limitation of the study. The “gold standard” serological test for leptospirosis recognised by the World Organisation for Animal Health (OIE) is MAT [44]. The MAT is, however, considered a relatively weak gold standard by most specialists in this field. The ELISA compares favourably to other published and commercial ELISAs based on sensitivity and specificity. The mean sensitivity and specificity, compared to the MAT test, for 10 published *Leptospira* Hardjo ELISAs is 96.31% and 90.62%, respectively [34,45-53]. It must be noted that the manufacturers of the LLK have changed the criteria for the calculation and interpretation of results since 2009, with S/P ratios now used. The current LLK has a declared sensitivity of 94.1% and a specificity of 94.8%. When the original OD values are used, the new S/P Ratio calculations yield an individual animal seroprevalence of 46% and a herd-level seroprevalence of 89.9% (using *FreeCalc* with the new values for ELISA sensitivity and specificity). The differences in herd-level and individual animal seroprevalence between the actual findings of this study, using the original calculations as detailed in the Materials and Methods, and the findings when using S/P ratios and the changes in test sensitivity and specificity would be statistically significant. However, there are material differences in the constituents of the current LLK compared to the LLK ELISA used in this study (peroxidase conjugated antibody of 1000 x concentrate versus the original peroxidase conjugated antibody of 5000 x concentrate; stop reagent of 1 M H2SO4 versus the original stop reagent of 0.5 M H2SO4). This means that it is not possible to validate a direct comparison of test results using both kits. It is the authors’ view that this does not invalidate the results of this study. Although the results obtained in this study may not be directly comparable to results generated according to the manufacturer’s current recommendations, neither are they directly comparable to results generated by other ELISA methods nor to results generated by the MAT, as published in other prevalence surveys.

Most tests have imperfect animal-level sensitivity and specificity, which means that the categorisation of the herd as either positive or negative (i.e. herd tests) is also imperfect [54]. To overcome the problem of relative lack of specificity of the LLK ELISA, and to account for the finite nature of the population, we used a published formula [38], and a within-herd seroprevalence of 40% was selected as indicative of herd infection. At a within-herd seroprevalence of 40%, it is likely that there are carrier animals in a herd and active transmission of leptospires.

### Interpretation

The finding of a national herd prevalence of *Leptospira* Hardjo of 82.29% (HSENS & HSPEC of 95%) suggests that bovine leptospirosis is endemic in the Irish suckler cattle population. This finding is closely aligned with the findings of Leonard et al. [29], who found that 79% of Irish dairy herds had a positive ELISA titre on bulk milk analysis. In work carried out in Northern Ireland in the early eighties, cultural and serological studies indicated that infection by the Hebdomadis serogroup was already highly prevalent in the Northern Ireland cattle population. In a combined random survey of both beef and dairy cattle, 34.7% had antibody titres of 1:100 or greater to serotype Hardjo using the MAT test, while leptospires belonging to the Hebdomadis serogroup were isolated from the kidneys of 57 (28.5%) of the cattle cultured [17]. The herd prevalence of *Leptospira* Hardjo appears to be higher in the Republic of Ireland than in many countries around the world: the serological herd prevalence of *Leptospira* Hardjo in beef herds in England was 72% [55]; in Spain, herd prevalence was 11% among beef herds [4]; and in the USA 42% of suckler herds had evidence of infection with *Leptospira borgpetersenii* serovar Hardjo*,*[56].

The high individual animal seroprevalence of 41.75% nationally, is also highly significant. Ellis and colleagues reported a high rate of Leptospiral carriage among heifers and aged cows in a Belfast abattoir in 1986 [57]. Following bacteriological culture, they found that 57% of animals had serovar Hardjo in their genital tracts and 62% in their urinary tracts [57]. Our prevalence findings are far in excess of published seroprevalence data from England, where animal seroprevalence figures of 24.2% [58] and 18% [55] have been described in mixed beef and dairy herd studies.

The potential factors contributing to the high herd and individual animal seroprevalence of *Leptospira* Hardjo in the Irish suckler cattle population are unknown. According to Ellis, strains such as Hardjo that are adapted to and maintained by cow-to-cow transmission are independent of region and rainfall [2]. It is most likely then, that the high herd and animal seroprevalence in this country is related to the high level of carrier animals [57] and standard suckler farming practices in Ireland that facilitate transmission of the disease, for example housing of cows and calves together over the winter period. In an Irish context, it appears that calves, reared alongside carrier cows, are exposed to Hardjo at a young age and are already seropositive prior to 12 months of age. This is in contrast to findings in epidemiological studies in dairy herds where heifers are much more likely to be immunologically naïve on entering the milking herd [59]. The increased sensitivity of recently developed ELISAs, such as the LLK ELISA, may also explain the increased animal seroprevalence found in this study.

The relationship between herd prevalence and breeding herd size in the same regions is a key result. The highest herd prevalence occurred in Region 5 (South-East), as did the largest median breeding herd size. Similarly, the lowest herd prevalence and the lowest median breeding herd size occurred in Region 2 (West). As with herd-level prevalence, the main factor associated with increased individual animal seroprevalence was breeding herd size, an association which was statistically significant (*P* < 0.001). The reason for this association most likely relates to the increased risk of exposure, transmission and persistence of infections in larger intensive herds [60,61]. A positive association between herd size and the presence of positive animals has been reported previously for Hardjo infection in cattle [12,62].

The regional variation in prevalence that was found in this study has been reported in other studies also: in Switzerland [63], Australia [64], Mexico [65,66] and the USA [57,67]. Collectively, those authors reported a range of possible factors for the regional differences, including soil type, mean temperature and herd management practices. However, almost all of these studies involved a number of Leptospiral serovars as well as Hardjo. As cattle are the maintenance host for *Leptospira* Hardjo, environmental influences such as soil type, rainfall and mean temperature are unlikely to contribute significantly to the regional variation in Hardjo prevalence in Ireland [2]. It is the view of the authors, based on the findings of this current study, that the high prevalence occurring in the South East of Ireland is related directly to the larger suckler herd sizes in this region with increased transmission of infection, as previously mentioned.

The finding of statistically significant differences in breed seroprevalence, especially between Aberdeen Angus and Belgian Blue, has not been described previously. However, due to the heterogenous nature of these breeds, care must be taken not to over-interpret these findings.

### Implications for the farming industry

The high prevalence of *Leptospira* Hardjo in Irish suckler herds may have implications for both animals and humans. There is recent evidence from a number of countries that Hardjo continues to cause substantial reproductive losses in cattle through abortion [11,68-73], and infertility [57,70,74-78]. Thus it is possible that losses in unvaccinated Irish suckler herds may be underestimated, although further work is required to establish the true extent of disease due to *L.* Hardjo in these herds. It will be important to relate the findings of this study to Irish suckler farmers, through educational bodies and bodies working towards the improvement of animal health and welfare throughout the country, e.g. Animal Health Ireland (AHI).

In addition to possible losses in these herds due to animal disease, zoonotic disease due to *Leptospira* Hardjo could also occur. There is a higher incidence of zoonotic disease due to *Leptospira* Hardjo in the Republic of Ireland than in England, with the highest incidence in the South-East [79]. It is thought that this increased incidence of disease due to Hardjo in the South-East is associated with the high concentration of cattle per square kilometre, both dairy and beef, in this region [79]. The same zoonotic risk does not apply in a suckler herd compared to a dairy herd. Dairy farmers are at most risk from urine splashing in the parlour. However, in a serological survey of farmers in Northern Ireland, antibodies to *L.* Hardjo were found in 2.9% of beef producers and 1.9% of mixed or arable farmers, as well as 6.4% of milk producers [80]. Therefore, suckler farmers and veterinary practitioners must continue to take correct precautions when calving suckler cows and when dealing with vaginal discharges.

## Abbreviations

AHI: Animal Health Ireland; ANOVA: Analysis of Variance; BHS: Breeding Herd Size; CMMS: Cattle Movement Monitoring System; ELISA: Enzyme Linked Immunosorbent Assay; HSENS: Herd-level Sensitivity; HSPEC: Herd-level Specificity; LLK: LinnodeeTM Bovine Leptospirosis Kit ELISA; MAT: Microscopic Agglutination Test; ml: Millilitre; nm: Nanometre; OD: Optical Density; OIE: World Organisation for Animal Health; P: Probability Value; UK: United Kingdom; USA: United States of America.

## Competing interests

None of the authors has any financial or personal relationships that could inappropriately influence or bias the content of the paper. This research was funded by Intervet/Schering Plough Animal Health, a pharmaceutical company that manufactures and sells a vaccine against *Leptospira* Hardjo. However, this has not influenced the nature of the study, the results of the study or the conclusions of the study.

## Authors’ contributions

EGR collected the serum samples, performed the ELISA tests and was the primary author of the paper. NL provided specific expertise in the field of bovine leptospirosis, as well as acting as one of the supervisors of the project. SM and LOG provided expertise in relation to the statistical interpretation of data and the structured writing of the paper, in addition to supervising the project. MLD acted as principal supervisor to the project and provided expertise in the area of population research. All authors read and approved the final manuscript.
